# Does Garden type I incomplete femoral neck fracture really exist in older adults? To evaluate the stability and consistency of Garden classification

**DOI:** 10.1186/s12893-022-01722-9

**Published:** 2022-07-15

**Authors:** Zhencun Cai, Zelin Zhang, Lixuan Ren, Chengzhe Piao, Liangbi Xiang

**Affiliations:** 1 Department of Orthopaedics Surgery, Central Hospital Affiliated to Shenyang Medical College, Liaoning Shenyang, China; 2 Department of Orthopaedics Surgery, General Hospital of Northern Theater Command, Shenyang, Liaoning China

**Keywords:** Femoral neck fracture, Garden classification, CT, X-ray

## Abstract

**Background:**

Accurate classification of femoral neck fracture (FNF) is crucial for treatment plan and therapeutic outcomes. Garden classification is commonly used in the clinic, but its stability and consistency remain controversial. The aim of this study was to evaluate the stability and consistency of Garden classification based on X and CT images, and to analyze whether it is valid for Garden I in the elderly.

**Methods:**

X-ray and CT images from 886 elderly patients with FNF were collected, four orthopaedic surgeons and four radiologists evaluated these images independently, and determined the fracture type based on Garden classification. Three months later, The exercise was repeated and the results were compared based on 4 types Garden classification (I, II, III and IV) and 3 types Garden classification (I + II, III and IV). Kappa was used to measure inter- and intraobserver agreement. The patients with Garden I incomplete FNF confirmed by 8 observers together based on images combined with medical history were compared with the intraoperative results.

**Results:**

Four types Garden classification, there was little consistency inter- and intraobservers (Kappa from 0.18 to 0.43) based on X-ray images, while professors consistency (0.56 to 0.76) was higher than residents (0.28 to 0.35) based on CT. 3 types Garden classification showed almost perfect agreement inter- and intraobservers, which ranged from 0.76 to 0.90. Totally 52 patients were diagnosed as Garden I, 38 of whom underwent arthroplasty. All surgical cases showed complete fracture during operation.

**Conclusions:**

There was low consistency and repeatability in 4 types Garden classification (I, II, III and IV), while 3 types Garden classification (I + II, III and IV) had high consistency among observers. In the elderly, all undisplaced femoral neck fracture may be Garden II, no Garden I.

## Introduction

The incidence rate of femoral neck fractures (FNF) is high, especially in the elderly. Totally 4.5 million elderly people worldwide become disabled due to hip fractures every year, including about 1.7 million patients with FNF [1, 2]. Such fractures are rarely treated conservatively, but usually treated by cannulated screw internal fixation, femoral head arthroplasty or total hip arthroplasty [3, 4]. The type of fractures is one of the important factors determining the treatment programs. Currently, orthopedic surgeons prefer hip arthroplasty for treating displaced FNF (Garden III and IV). However, the treatment of incomplete FNF or undisplaced FNF (Garden I and II) still remains controversial [5, 6]. Most scholars believe that Garden I is incomplete FNF, and can be treated by cannulated screw internal fixation [5, 7], while there is much controversy over the treatment of Garden II in the elderly. Some scholars believe that Garden II in the elderly can be treated with cannulated screw internal fixation [8], while some recommend hip arthroplasty because Garden II is complete FNF and osteoporosis exits in the elderly, which result in high rate of nonunion and femoral head necrosis after internal fixation [9, 10]. Studies have shown that in the treatment of FNF in the elderly by internal fixation, the reoperation rate is > 10% [11]. Therefore, the accurate classification of FNF in the elderly is very important for both choosing of treatment programs and achieving satisfactory outcomes .

Garden classification, a commonly used classification method for FNF in the clinic, was proposed by Professor RS Garden in 1961 [12, 13]. In spite of arguments, Garden classification is still the most widely used FNF classification method so far [14, 15]. RS Garden classified FNF into four types based on completeness and displacement degree of FNF: Garden I is defined as incomplete and undisplaced FNF, Garden II as complete and undisplaced FNF, Garden III as complete and partially-displaced FNF, and Garden IV as complete and completely-displaced FNF. The significance of Garden classification is to judge the severity of fractures and predict the correlation between FNF and femoral head necrosis. Both FNF severity and incidence of femoral head necrosis of Garden I–IV FNF increase in sequence. However, it is difficult to judge Garden classification clinically, especially between Garden I and II [16], and some scholars even speculate that Garden I does not actually exist [17, 18].

Garden classification is mainly based on the plain radiographs generated by radiographic examination [19]. X-ray, characterized by low price and easy operation, is also the first choice for fracture examination. However, due to the location particularity of FNF, structural overlap of femoral neck itself, and the difficulty of the patients with FNF to fully cooperate in postural examination and other factors, clinically, only the anterior–posterior radiographs of hips can be taken, which cannot truly and objectively reflect fracture characteristics to easily result in misdiagnosis and missed diagnosis [20, 21]. Therefore, there are certain errors in fracture classification based on radiographs. Previous studies have shown that even from the same FNF X-ray, different observers may draw different classification conclusions and the same observer may draw different conclusions when making classification at different times [16]. However, CT can effectively improve the identification accuracy of FNF Garden classification because CT examination is less affected by postures, and through thin-slice reconstruction, VR, MPR and other processing, CT can effectively reconstruct the 3D image of femoral neck and clearly and intuitively display the changes of bone cortex [22]. However, whether the accuracy and repeatability of classification based on CT data are really better than that based on plain radiographs is also controversial [23].

The aim of this study was to evaluate the stability and consistency of Garden classification based on X and CT images, and to analyze whether it is valid for Garden I in the elderly.

## Methods

### Subjects

Totally 886 FNF patients admitted to the Affiliated Central Hospital of Shenyang Medical College from June 2018 to June 2021 were included in this study. Institution’s ethic board approved the investigation (No. 20210918) Written informed consent was obtained from the patients for publication of this study and any accompanying images.

Inclusion criteria: (1) older than 65 years, (2) had a clear history of trauma; (3) suffered from fresh unilateral FNF, without fractures in other parts; (4) had complete plain radiographs and CT examination data; (5) had complete surgical records and other treatment materials. Exclusion criteria: (1) the patients had pathological fractures such as bone tumor and bone tuberculosis; (2) the patients had old fractures or fractures at other parts besides fresh unilateral FNF; (3) the patients had surgical contraindications such as severe nerve and vascular injury and coagulation dysfunction.

### Examination

#### X-ray examination

All FNF patients were kept in supine position, with their both lower limbs in 15° of internal rotation; digital radiography system (DR) (Germany Siemens DR240) was used to take standard anteroposterior and lateral radiographs of pelvis, with the parameters set as follows: the anteroposterior central line pointed to the midpoint of superior margin of pubic symphysis and bilateral anterior superior iliac spine connection, the focus-film distance was 1.2 m, the tube current was 160 mAs, and the voltage was 65 kV.

#### CT 3D examination

All of the elderly FNF patients were kept in supine position; spiral CT machine (Germany Siemens 128-row spiral CT machine) was used to first perform routine scanning from the superior border of ilium to pubic symphysis, and then perform 2 mm thin-slice reconstruction, volume reconstruction (VR), multiplanar reconstruction (MPR) and other processing on the original data; the angle and image quality parameters were adjusted reasonably according to clinical requirements to obtain the 3D structure images of hip, femoral head and femoral neck. During the examination, the parameters were set as follows: tube voltage was 130 kV, tube current was 50 mAs, and slice thickness was 2 mm.

### Classification

Step 1: The patient information was removed from the X-ray image data of the 886 patients, and these X-ray image data were then randomly numbered; Four radiologists (i.e. two professors who have been working for more than 15 years A and B, and two residents C and D) and four orthopedic surgeons (i.e. two professors who have been working for more than 15 years E and F, and two residents G and H) were arranged to perform Garden classification; All observers were familiar with Garden classification criteria, and they observed the trend, location and soft tissue injury of fracture line by reading X-ray radiographs separately to complete Garden classification for FNF. Step 2: The patient information was removed from the CT image data of the 886 patients, and these CT image data were then randomly numbered; The above-mentioned 8 physicians once again made judgment on the CT image data separately and completed Garden classification for FNF. All judgments were recorded and then deposited in the database that was managed by the main investigator. Step 3: Three months later, the above-mentioned 8 physicians once again read the image data separately to complete Garden classification for the X-ray and CT images respectively. The results were compared based on 4 types Garden classification (I, II, III and IV) and 3 types Garden classification (I + II, III and IV). Step 4: After the separate classification, the 8 observers reviewed the image data of the 886 patients together. The X-ray and CT image data and the medical history like the cause of injury before treatment etc. of all the patients were available for consultation. The 8 observers combined X-ray images and CT images to jointly carry out the classification and find out Garden I cases. For the Garden I cases the 8 observers found out jointly, the surgical records and intraoperative photos were consulted to compare and analyze the consistency between intraoperative findings and imaging data.

### Statistical analysis

Statistical analysis was performed by calculating the Cohen kappa value using SPSS 23.0 statistical software for inter- and intraobserver reliability as observed agreement. To calculate the multi-rater kappa for the interobserver agreement the statistical method of Fleiss was used [24]. We interpreted the kappa value coefficient according to the guidelines proposed by Landis and Koch: less than 0.00 poor reliability, 0.00 to 0.20 slight reliability, 0.21 to 0.40 fair reliability, 0.41 to 0.60 moderate reliability, 0.61 to 0.80 substantial agreement and 0.81 to 1.00 almost perfect agreement [25]. For additional calculations, Microsoft Excel 2018 was used.

## Results

Totally 886 FNF patients were included in this study, including 290 males and 596 females, aged 65–96 years. Causes of injury: there were 258 road traffic injury cases, 126 fall injury cases, and 502 tumble injury cases. Duration of visit was 1–9 h, with an average of 4.82 h. There were 503 cases of left FNF and 383 cases of right FNF.

Two times of Garden classification based on X-ray were performed, and the classification results by the 8 observers are shown in Table 1. Consistency test based on 4 types Garden classification (I, II, III and IV): Kappa values among professors (A, B, E and F) ranged from 0.24 to 0.43, and the values among residents (C, D, G and H) ranged from 0.18 to 0.35. The Kappa values between the professors ranged from 0.38 to 0.42, and the values between residents ranged from 0.26 to 0.36. There was little consistency inter- and intraobservers (Table 2).

**Table 1:** Results of classification according X-ray images

Type	Observer							
	A	B	C	D	E	F	G	H
I	106 (89)	85 (112)	116 (81)	93 (128)	128 (77)	109 (93)	68 (133)	126 (103)
II	175 (197)	203 (186)	170 (224)	192 (169)	162 (219)	186 (199)	223 (171)	171 (185)
III	336 (345)	343 (327)	359 (348)	329 (335)	344 (325)	313 (339)	327 (345)	330 (319)
IV	268 (255)	255 (261)	241 (233)	252 (254)	252 (265)	278 (235)	268 (257)	259 (279)

Second observations is in brackets

**Table 2:** Kappa values for the 4 types Garden classification of X-ray images

Type	Interobserver								Intraobserver
	A*	B*	C	D	E*	F*	G	H	
A*	X								0.39
B*	0.40	X							0.41
C	0.21	0.30	X						0.26
D	0.29	0.32	0.18	X					0.31
E*	0.38	0.24	0.27	0.33	X				0.38
F*	0.35	0.29	0.36	0.31	0.43	X			0.42
G	0.20	0.23	0.31	0.25	0.31	0.36	X		0.36
H	0.19	0.38	0.29	0.24	0.32	0.24	0.35	X	0.28
Mean	0.37	0.35	0.26	0.28	0.33	0.30	0.25	0.21	0.36

A, B, C and D are radiologists, E, F, G and H are orthopedic surgeons; * are professors

The Garden classification results concluded by the 8 observers according to CT image data are shown in Table 3. Consistency test based on 4 types Garden classification: The Kappa values among and between the professors ranged from 0.56 to 0.76, and the values among and between the residents ranged from 0.28 to 0.35. There was higher consistency among and between the professors (Table 4). The number of Garden I evaluated by each observer according to CT was less than that evaluated according to X-ray. and the number of Garden II according to CT was more than X-ray; however, the total number of Garden I and II evaluated according to CT was basically the same as X-ray.

**Table 3:** Results of classfication according CT images

Type	Observer							
	A	B	C	D	E	F	G	H
I	86 (75)	72 (82)	95 (69)	75 (108)	66 (73)	85 (93)	60 (96)	100 (80)
II	193 (209)	216 (196)	195 (229)	213 (183)	218 (220)	206 (199)	226 (195)	193 (216)
III	341 (349)	350 (339)	349 (353)	336 (342)	343 (336)	335 (339)	342 (354)	348 (329)
IV	266 (253)	248 (269)	247 (235)	262 (253)	259 (257)	260 (255)	258 (241)	245 (261)

Second observations is in brackets

**Table 4:** Kappa values for the 4 types Garden classification of CT images

Type	Interobserver								Intraobserver
	A*	B*	C	D	E*	F*	G	H	
A*	X								0.69
B*	0.67	X							0.73
C	0.35	0.38	X						0.36
D	0.39	0.31	0.35	X					0.37
E*	0.76	0.68	0.42	0.40	X				0.58
F*	0.69	0.56	0.38	0.36	0.59	X			0.70
G	0.33	0.36	0.32	0.31	0.31	0.25	X		0.35
H	0.29	0.44	0.29	0.28	0.32	0.25	0.30	X	0.29
Mean	0.58	0.46	0.36	0.33	0.56	0.43	0.39	0.31	0.45

A, B, C and D are radiologists, E, F, G and H are orthopedic surgeons; * are professors

The classification results of Garden I and II were combined in one group to perform the consistency test again. Kappa values in 3 types Garden classification (I + II, III and IV) showed almost perfect agreement inter- and intraobservers, which ranged from 0.76 to 0.90 (Table 5). regardless of whether Garden classification was based X-ray or CT

**Table 5:** Kappa values for the 3 types Garden classification of CT and X -ray images

Type	Interobserver								Intraobserver
	A*	B*	C	D	E*	F*	G	H	
A*	X								0.89 (0.83)
B*	0.90 (0.85)	X							0.90 (0.85)
C	0.89 (0.80)	0.88 (0.86)	X						0.87 (0.84)
D	0.88 (0.84)	0.82 (0.80)	0.86 (0.82)	X					0.88 (0.82)
E*	0.90 (0.85)	0.89 (0.81)	0.87 (0.84)	0.83 (0.80)	X				0.86 (0.81)
F*	0.86 (0.83)	0.87 (0.77)	0.82 (0.78)	0.89 (0.80)	0.88 (0.82)	X			0.87 (0.87)
G	0.80 (0.77)	0.86 (0.76)	0.87 (0.81)	0.89 (0.88)	0.88 (0.83)	0.77 (0.79)	X		0.89 (0.82)
H	0.89 (0.82)	0.82 (0.79)	0.85 (0.78)	0.88 (0.86)	0.90 (0.85)	0.84 (0.88)	0.86 (0.77)	X	0.84 (0.81)
Mean	0.87 (0.82)	0.85 (0.80)	0.82 (0.79)	0.84 (0.80)	0.89 (0.78)	0.88 (0.86)	0.86 (0.83)	0.83 (0.80)	0.88 (0.81)

A, B, C and D are radiologists, E, F, G and H are orthopedic surgeons; * are professors. Values of X -ray is in brackets

**Fig. 1:**
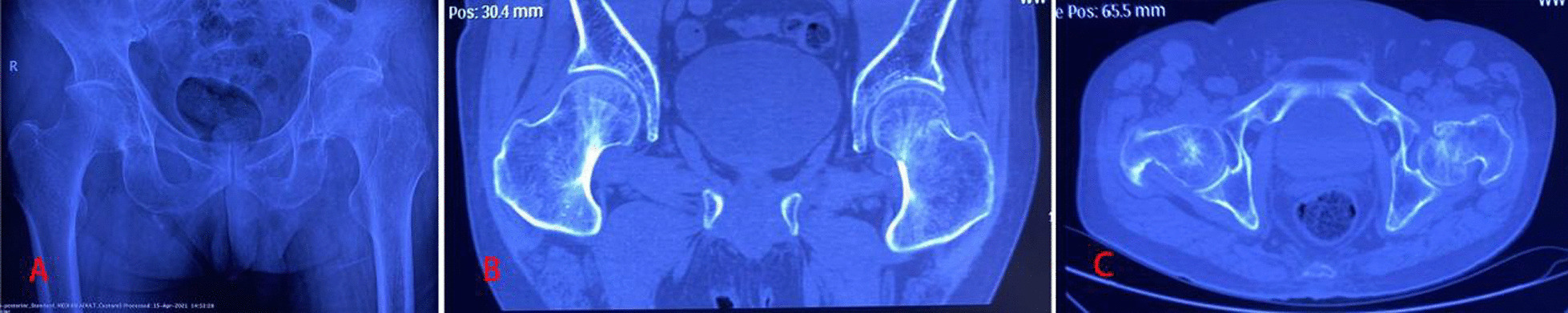
Case 1 of a 68 years old male patient with left FNF. **A** Classified as Garden I, i.e. incomplete FNF, only involving lateral cortex break, with medial cortex remaining intact, as shown on X-ray. **B** Classified as Garden II, i.e. complete FNF, involving medial cortex break, as shown on CT coronal plane. **C** Classified as Garden II, i.e. complete FNF, involving slight displacement, as shown on CT horizontal plane

**Fig. 2:**
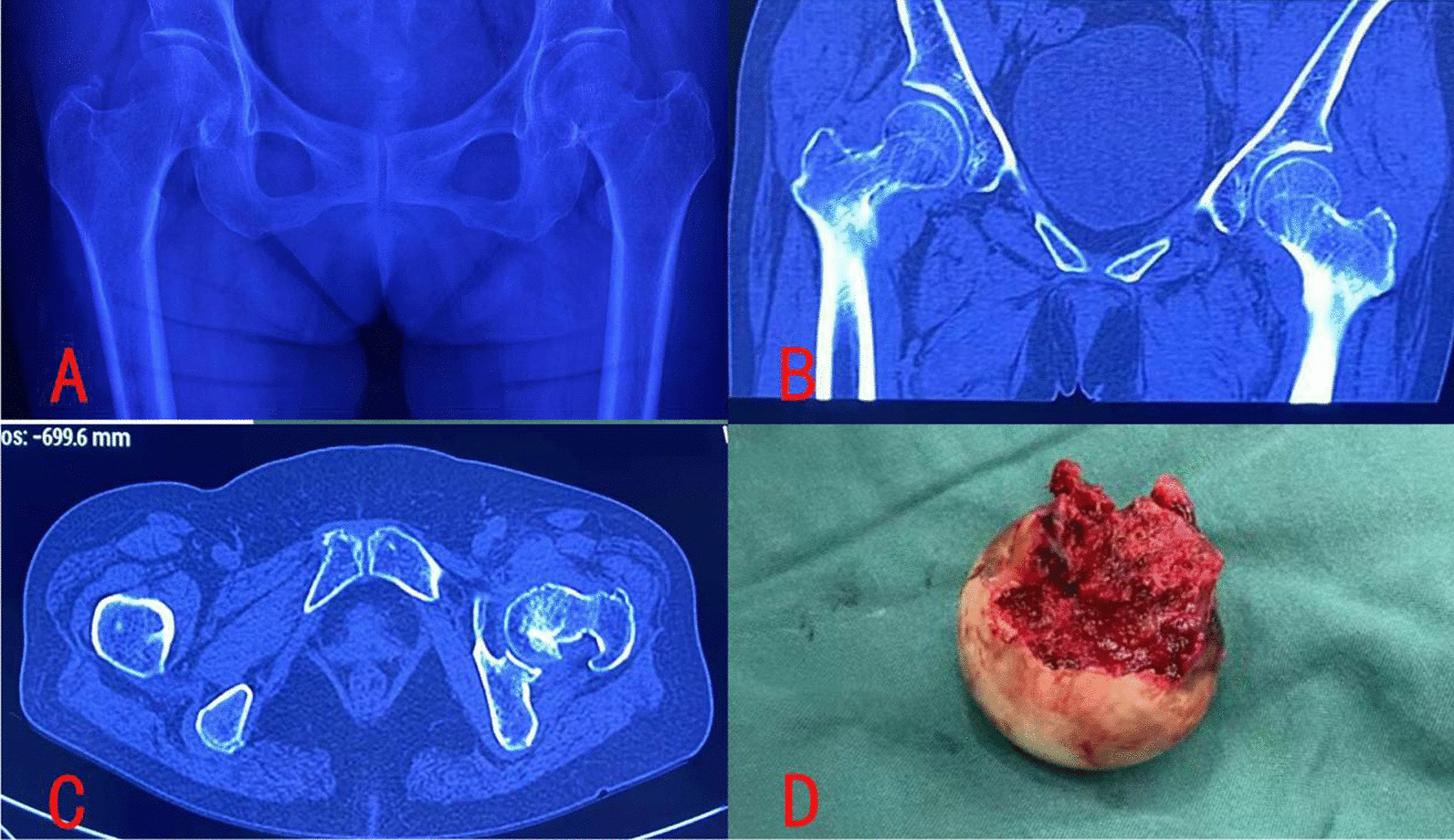
Case 2 of a 70 years old female patient with left FNF. **A** Classified as Garden I, i.e. incomplete FNF, only involving medial cortex break, with lateral cortex remaining intact, as shown on X-ray. **B** and **C** Classified as Garden I, i.e. incomplete FNF, with lateral cortex remaining intact, as shown on CT coronal plane and horizontal plane. **D** Classified as Garden II, i.e. complete FNF, as found during operation

After the 8 observers jointly reviewed the X-ray and CT imaging data, 52 patients were identified as Garden I. According to the surgical records and case data of patients, 14 patients were treated by closed reduction and cannulated screw internal fixation, and 38 patients were treated by artificial femoral head replacement or total hip replacement. For the patients treated by closed reduction, the real situation of their FNFs could not be identified. However, for the 38 patients treated by artificial hip replacement, it can be seen that their FNFs were complete FNFs (intraoperative findings): 22 cases of FNFs had anterior-posterior displacement or valgus impacted fractures, and only 16 cases of FNFs were undisplaced fractures, whose fracture lines also completely involved the lateral and medial cortex (Figs. 1 and 2).

## Discussion

The incidence rate of FNF accounts for about 50% of hip fractures, most commonly in the elderly patients with osteoporosis. The incidence of FNF is mainly unilateral. The number of patients over 50 years old accounts for about 74.0% of the total number of hip fracture patients [26]. FNF in the elderly is mostly caused by low-energy trauma such as accidental falls, body twisting and falling down to the ground. With the acceleration of population aging, the incidence of FNF is also increasing rapidly. There are many clinical treatment programs for FNF, but there is still a lack of unified treatment standard [27]. Non-operative treatment often requires long-term bedridden, which leads to an increase in the incidence of complications such as pneumonia, bedsore and deep venous thrombosis in the elderly patients [5], so many elderly patients with FNF often die within half a year after non-operative treatment because of complications. Even if surgical treatment is adopted, there are still following problems: fracture nonunion and femoral head necrosis may occur after cannulated screw internal fixation; artificial hip replacement can quickly restore the walking function of patients, but there are also risks of prosthesis loosening and infection, and the artificial hip has a limited service life due to wear [28]. Therefore, FNF in the elderly is a disease that seriously affects the quality of life of patients. How to effectively treat FNF and improve the prognosis of patients is a great challenge the clinicians are facing.

The preoperative classification for FNF directly affects the prognosis of patients to a great extent. The type of fractures reflects the severity of the disease and determines the treatment programs. At present, the most widely used FNF classification method is Garden classification based on the fracture line characteristics and displacement degree: Garden I and II FNF are undisplaced FNF; Garden III and IV FNF are displaced FNF; displacement degree of Garden I–IV increase in sequence [15]. In recent years, many doctors have found out some shortcomings in Garden classification in clinical application [15]. Frandsen et al. [29] have asked 8 doctors to perform separate Garden classification for 100 cases of FNF. It was found that the interobserver coincidence rate in the classification results by the 8 doctors was only 22%, and the number of cases in controversial over existence of displacement accounted for 33%. Embden et al. [16] have carried out Garden classification for 100 cases of FNF, inter-observer kappa for the Garden classification was only 0.31. It can be seen that the judgment for displacement in Garden classification is closely related to subjective factors. Garden III and IV are displaced FNF. It is easy to distinguish between Garden III and Garden IV according to the degree of displacement, and at present, the clinical diagnosis for them is relatively unified. However, many studies have shown that it is difficult to distinguish between Garden I and Garden II because it is difficult to judge whether an undisplaced FNF is complete or incomplete FNF [16, 18].

What we found is that, both senior professors and junior residents had obvious inconsistencies in the identification of Garden I and II. Even with the high-resolution CT image data, the 4 junior residents still had significant inconsistency in identification of Garden I and II. If the same observer reads the same X-ray or CT image again after an interval of time, he may also draw an inconsistent conclusion. These results show that, it is indeed difficult to distinguish between Garden I and Garden II in the elderly by X-ray radiographs, which is irrelevant to the qualifications, age and experience of the observers. The coincidence rate in the classification results concluded by senior professors according to the CT image is relatively high. Therefore, it is suggested that, for undisplaced FNF, senior experienced doctors should be arranged to read CT image data to determine the classification type and treatment programs.

Besides, in this study, we found that although there were obvious inconsistencies in the identification results of Garden I and II among the observers, there was no significant difference in the identification results of Garden I and II among the observers when the consistency test was re-performed by combining the classification results of Garden I and II in one group. In other words, although it is difficult to distinguish between Garden I and Garden II, it is relatively easy to distinguish Garden I and II from Garden III and IV. Beimers et al. [18] suggested that, in order to facilitate accurate classification, FNF can be simply classified into undisplaced type (Garden I and II) and displaced type (Garden III and IV). Through a questionnaire survey on 298 orthopedic surgeons, Bhandari et al. [30] found that, out of the 298 orthopedic surgeons surveyed, 72% thought Garden classification is the preferred FNF classification method, but only 39% thought they can tell Garden I, II, III and IV apart, while 96% thought they can distinguish between undisplaced FNF (Garden I and II) and displaced FNF (Garden III and IV). In our study, we draw the same conclusion as theirs. However, we do not recommend not distinguishing between Garden I and Garden II, because distinguishing between Garden I and Garden II is very important for guiding the treatment.

Garden I is defined as incomplete FNF, involving partial cortical fracture. In recent years, some people believe that valgus impacted FNF is undisplaced and stable fracture, also classified as Garden I. However, many people doubt the existence of Garden I (incomplete FNF, involving partial cortical fracture) [17, 18]. Chen et al. [17] prospectively studied the imaging data of 825 FNF cases and confirmed that the Garden I shown on X-ray were actually complete FNF by CT scanning. We further combined the imaging data (X-ray and CT data) with the surgical findings, and got the same conclusion as Chen’s. Based on the X-ray and CT data, our 8 observers jointly confirmed that the number of Garden I cases was 52, of which 38 cases were treated with artificial hip replacement. During the surgery, we intuitively found that these 38 Garden I cases were actually complete FNF, with circular fracture line, involving lateral and medial cortex, and out of them, 22 cases had anterior-posterior displacement or valgus impacted fractures. Therefore, in the elderly, Garden I incomplete FNF may not exist. The elderly have decreased bone mineral density and osteoporosis, so their femoral necks are prone to fracture once they are subjected to external force [31]. Besides, the bones of the elderly have the features of decreased elasticity and enhanced brittleness, and the anatomical position of femoral neck is the turning part of force line [32]. Therefore, in case of FNF in the elderly, it is difficult to have partial cortical fracture, but more likely to have complete fracture. Complete FNF in the elderly may be presented as incomplete FNF on X-ray due to the influence of fracture anatomical structure and projection angle.

The limitation of this study is that the FNF patients selected for this study are the elderly. There may be real Garden I FNF in adolescents or children. We will further conduct a large sampled study.

## Conclusions

Garden classification is the most popular classification system for FNF. There was low consistency and repeatability in 4 types Garden classification (I, II, III and IV) among different observers, while 3 types Garden classification (I + II, III and IV) had high consistency among observers. When performing Garden classification for FNF, the X-ray, CT and medical history of the patients all should be combined as classification basis. The undisplaced FNF in the elderly can only be Garden II, unlikely to be Garden I.

## Data Availability

The datasets generated and/or analysed during the current study are not publicly available due to limitations of ethical approval involving the patient data and anonymity but are available from the corresponding author on reasonable request.
